# Species of *Dendrostoma* (Erythrogloeaceae, Diaporthales) associated with chestnut and oak canker diseases in China

**DOI:** 10.3897/mycokeys.48.31715

**Published:** 2019-03-06

**Authors:** Ning Jiang, Xin-Lei Fan, Pedro W. Crous, Cheng-Ming Tian

**Affiliations:** 1 The Key Laboratory for Silviculture and Conservation of the Ministry of Education, Beijing Forestry University, Beijing 100083, China Beijing Forestry University Beijing China; 2 Westerdijk Fungal Biodiversity Institute, Uppsalalaan 8, 3584 CT, Utrecht, The Netherlands Westerdijk Fungal Biodiversity Institute Utrecht Netherlands

**Keywords:** Canker, *
Castanea
*, multi-gene phylogeny, *
Quercus
*, systematics

## Abstract

*Dendrostoma* was recently proposed in Erythrogloeaceae (Diaporthales, Sordariomycetes), with all known members recorded as being plant pathogenic on economically important tree hosts. During our collections of *Dendrostoma* species in China, mild to severe canker symptoms were observed on sweet chestnut (*Castaneamollissima*) and oak (*Quercus* spp.) trees. Dead and dying plant tissues exhibiting Dendrostoma canker symptoms were sampled for fungal isolation. A total of 37 *Dendrostoma* isolates were obtained and analysed using morphological characteristics and molecular data (ITS, LSU, *RPB2*, *TEF1-α*). Based on these data, 10 novel clades could be distinguished, which also proved to represent morphologically distinct species described here as *Dendrostomaaurorae*, *D.castaneae*, *D.castaneicola*, *D.chinense*, *D.dispersum*, *D. parasiticum*, *D.qinlingense*, *D.quercus*, *D.shaanxiense* and *D.shandongense* spp. nov. A key to species of the genus is also provided.

## Introduction

The family Erythrogloeaceae was established to accommodate *Chrysocrypta*, *Disculoides*, and *Erythrogloeum*, which exhibit epiphyllous acervuli along with subcylindrical to ampulliform conidiogenous cells and aseptate conidia (Senanayake et al. 2017). *Erythrogloeum* (Petrak 1953) is the type genus of Erythrogloeaceae and causes severe anthracnose on *Hymenaeacourbaril* in South America (Ferreira et al. 1992). *Chrysocrypta* was first proposed in Cryphonectriaceae, being associated with leaf spots on *Corymbia* spp. in Australia (Crous et al. 2012a), but was subsequently transferred to Erythrogloeaceae, based on DNA sequence data (Senanayake et al. 2017). *Disculoides* was introduced with two initial species, *D.eucalypti* and *D.eucalyptorum*, discovered on diseased *Eucalyptus* leaves in Australia (Crous et al. 2012b). Two additional *Disculoides* species, *D.calophyllae* and *D.corymbiae*, were subsequently reported as foliar pathogens of *Corymbiacalophylla* (Crous et al. 2016, 2017).

*Dendrostoma* (Erythrogloeaceae, Diaporthales) was recently introduced as a phytopathogenic fungal genus causing canker diseases on several economic hardwoods such as *Malusspectabilis*, *Osmanthusfragrans* and *Quercusacutissima* (Fan et al. 2018). Subsequently, *Dendrostomaleiphaemia* on *Quercus* trees was transferred from *Amphiporthe* based on ITS and LSU sequences analysis (Senanayake et al. 2018). *Dendrostoma* represents one of four genera in the family, but is the only one known to have a sexual morph. Hence, Erythrogloeaceae can be distinguished from the other diaporthalean families by multiguttulate and bicellular ascospores that are constricted at the septum and acervular conidiomata, with subcylindrical to ampulliform conidiogenous cells and hyaline to olivaceous, aseptate conidia (Rossman et al. 2007, Voglmayr and Jaklitsch 2014, Senanayake et al. 2017, Voglmayr et al. 2017, Fan et al. 2018).

The Erythrogloeaceae, including *Chrysocrypta*, *Dendrostoma*, *Disculoides* and *Erythrogloeum*, represent a family of fungal pathogens occurring on several commercially important tree genera such as *Corymbia*, *Eucalyptus*, *Hymenaea*, *Malus*, *Osmanthus* and *Quercus* in Australia, Brazil, China and Costa Rica (Petrak 1953, Ferreira et al. 1992, Crous et al. 2012a, b, 2016, 2017, Fan et al. 2018). Considering the importance of these tree diseases and the lack of taxonomic information on *Dendrostoma*, we conducted several surveys for members of the genus in China.

The aims of present study were (i) to describe the important *Dendrostoma* spp. associated with canker diseases on chestnut and oak trees in China and (ii) to provide a multi-gene phylogeny for the genus *Dendrostoma* based on a large set of freshly collected specimens in China. In agreement with previous taxonomic studies in Erythrogloeaceae, where different *Disculoides* spp. were discovered on Myrtaceae (Crous et al. 2012a, b, 2016, 2017), several *Dendrostoma* spp. were found on Fagaceae (*Castanea* and *Quercus*), being associated with mild to severe canker diseases. The *Dendrostoma* species were subsequently classified based on morphological characteristics and phylogenetic data.

## Materials and methods

### Sample collections and fungal isolates

**Figure 1.**
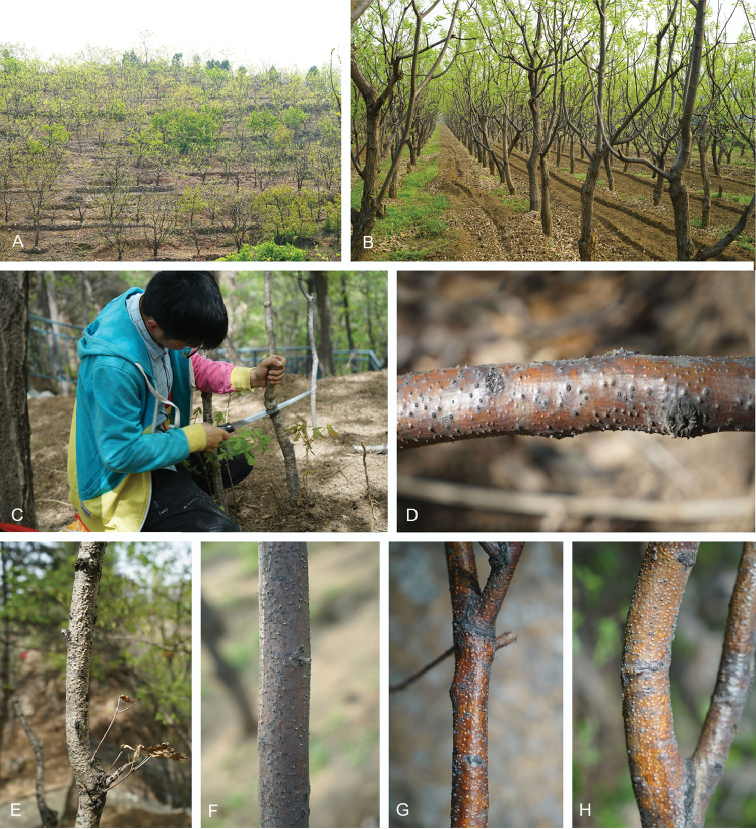
Chestnut plantations and Dendrostoma canker symptoms. **A** A chestnut plantation on the mountain **B** A chestnut plantation on the plain **C** Collection of the dead trees killed by *Dendrostoma* pathogens **D–H**Dendrostoma canker symptoms on host branches.

Surveys for *Dendrostoma* species were conducted in plantations, nurseries, parks, gardens, on mountains and natural reserves in Beijing, Hebei, Shaanxi, Shandong, Tianjin and Zhejiang Provinces in China from 2017 to 2018. Typical canker symptoms were observed on stems, branches and twigs of different hosts, including *Castaneamollissima*, *Quercusaliena*, Q.alienavar.acuteserrata, *Q.wutaishanica* and other *Quercus* species (Fig. 1). Diseased samples were collected and placed in paper bags, then transferred to the laboratory for further study.

A total of 37 *Dendrostoma* isolates were established by removing a mucoid spore mass from sporulating ascomata and conidiomata produced on diseased bark, spreading the suspension on the surface of potato dextrose agar (PDA) plates and incubating the plates at 25 °C in the dark for up to 24 h. Single germinating spores were then transferred to clean plates under a dissecting microscope with a sterile needle. Specimens and isolates were deposited in the Museum of Beijing Forestry University (BJFC). Axenic cultures are maintained in the China Forestry Culture Collection Centre (CFCC).

### Morphological analysis

The identification of *Dendrostoma* spp. was based on morphological features observed on the natural substrates. Cross-sections for ascomata and conidiomata from tree barks were prepared by hand using a double-edged blade under a dissecting microscope. At least 10 conidiomata/ascomata, 10 asci and 50 conidia/ascospores were measured to calculate the mean size and standard deviation. Measurements are reported as maxima and minima in parentheses and the range representing the mean plus and minus the standard deviation of the number of measurements is given in parentheses (Voglmayr et al. 2017). Microscopy photographs were captured with a Nikon Eclipse 80i compound microscope equipped with a Nikon digital sight DS-Ri2 high definition colour camera, using differential interference contrast illumination. Nomenclatural novelties and descriptions were deposited in MycoBank (Crous et al. 2004). Cultural characteristics were recorded for isolates incubated on PDA in the dark at 25 °C.

### DNA extraction, PCR amplification and sequencing

Genomic DNA was extracted from fungal colonies growing on PDA using a modified cetyl trimethyl ammonium bromide method (CTAB; Doyle and Doyle 1990, Zhang et al. 2010). The ITS region was amplified using the primers ITS1 and ITS4 (White et al. 1990), the LSU region with the primers LR0R and LR5 (Vilgalys and Hester 1990), the *RPB2* region with primers fRPB2-5F and fRPB2-7cR (Liu et al. 1999) and the partial *TEF1-α* gene with the primers EF1-728F and EF1-986R (Carbone and Kohn 1999). The PCR mixture for all regions consisted of 1 μl genomic DNA, 3 mM MgCl_2_, 20 μM of each dNTP, 0.2 μM of each primer and 0.25 U rTAQ DNA polymerase (TaKaRa, Shiga). Amplification of LSU and ITS were accomplished by an initial step of 2 min at 95 °C, followed by 35 cycles of 30 s at 95 °C, 30 s at 51 °C and 40 s at 72 °C, with a final extension of 10 min at 72 °C. For *TEF1-α* amplification, the 35 cycles consisted of initiation at 95 °C for 8 min, denaturation at 95 °C for 15 s, annealing at 55 °C for 20 s, elongation at 72 °C for 1 min and a final extension of 5 min at 72 °C. For *RPB2*, amplification of 35 cycles consisted of initiation at 95 °C for 5 min, denaturation at 95 °C for 30 s, annealing at 52 °C for 1 min, elongation at 72 °C for 1 min and a final extension of 10 min at 72 °C. The DNA sequencing was performed using an ABI PRISM 3730XL DNA Analyzer with BigDye Terminater Kit v. 3.1 (Invitrogen, Carlsbad) at the Shanghai Invitrogen Biological Technology Company Limited (Beijing).

### Phylogenetic analyses

Sequences generated from the above primers of the different genomic regions (ITS, LSU, *TEF1-α* and *RPB2*) were analysed in comparison with those of *Dendrostomamali* (CFCC 52102), *D.leiphaemia* (CBS 187.37), *D.osmanthi* (CFCC 52106, CFCC 52107, CFCC 52108 and CFCC 52109) and *D.quercinum* (CFCC 52103, CFCC 52104 and CFCC 52105) from Fan et al. (2018) and Senanayake et al. (2018). *Corymbiacorymbiae* (CBS 132528), *Disculoideseucalypti* (CBS 132183) and *D.eucalyptorum* (CBS 132184) were selected as the outgroup taxa (Crous et al. 2012a, b). All sequences were aligned using MAFFT v. 6 (Katoh and Toh 2010) and edited manually using MEGA v. 6 (Tamura et al. 2013). Phylogenetic analyses were performed using PAUP v. 4.0b10 for maximum parsimony (MP) analysis (Swofford 2003) and PhyML v. 3.0 for Maximum Likelihood (ML) analysis (Guindon et al. 2010). The first analyses were performed on the combined multi-gene dataset (ITS, LSU, *TEF1-α* and *RPB2*) to compare isolates of Erythrogloeaceae species to ex-type sequence data from recent studies (Table 1).

**Table 1.** Isolates and GenBank accession numbers used in the phylogenetic analyses.

Species	Culture	Location	Host	Host family	GenBank accession numbers			
					ITS	LSU	*TEF1−a*	*RPB2*
* Chrysocrypta corymbiae *	CBS 132528*	Australia	*Corymbia* sp.	Myrtaceae	JX069867	JX069851	MH545457	MH545415
* Dendrostoma aurorae *	CFCC 52753*	China	* Castanea mollissima *	Fagaceae	MH542498	MH542646	MH545447	MH545405
	CFCC 52754	China	* Castanea mollissima *	Fagaceae	MH542499	MH542647	MH545448	MH545406
* Dendrostoma castaneae *	CFCC 52745*	China	* Castanea mollissima *	Fagaceae	MH542488	MH542644	MH545437	MH545395
	CFCC 52746	China	* Castanea mollissima *	Fagaceae	MH542489	NA	MH545438	MH545396
	CFCC 52747	China	* Castanea mollissima *	Fagaceae	MH542490	NA	MH545439	MH545397
	CFCC 52748	China	* Castanea mollissima *	Fagaceae	MH542491	NA	MH545440	MH545398
	CFCC 52749	China	* Castanea mollissima *	Fagaceae	MH542492	MH542645	MH545441	MH545399
	CFCC 52750	China	* Castanea mollissima *	Fagaceae	MH542493	NA	MH545442	MH545400
	CFCC 52751	China	* Castanea mollissima *	Fagaceae	MH542494	NA	MH545443	MH545401
	CFCC 52752	China	* Castanea mollissima *	Fagaceae	MH542495	NA	MH545444	MH545402
* Dendrostoma castaneicola *	CFCC 52743*	China	* Castanea mollissima *	Fagaceae	MH542496	NA	MH545445	MH545403
	CFCC 52744	China	* Castanea mollissima *	Fagaceae	MH542497	NA	MH545446	MH545404
* Dendrostoma chinense *	CFCC 52755*	China	* Castanea mollissima *	Fagaceae	MH542500	MH542648	MH545449	MH545407
	CFCC 52756	China	* Castanea mollissima *	Fagaceae	MH542501	MH542649	MH545450	MH545408
	CFCC 52757	China	* Castanea mollissima *	Fagaceae	MH542502	MH542650	MH545451	MH545409
	CFCC 52758	China	* Castanea mollissima *	Fagaceae	MH542503	MH542651	MH545452	MH545410
* Dendrostoma dispersum *	CFCC 52730*	China	*Quercus* sp.	Fagaceae	MH542467	MH542629	MH545416	MH545374
	CFCC 52731	China	*Quercus* sp.	Fagaceae	MH542468	MH542630	MH545417	MH545375
* Dendrostoma leiphaemia *	CBS 187.37	NA	*Quercus* sp.	Fagaceae	MH855882	MH867393	NA	NA
* Dendrostoma mali *	CFCC 52102*	China	* Malus spectabilis *	Rosaceae	MG682072	MG682012	MG682032	MG682052
* Dendrostoma osmanthi *	CFCC 52106*	China	* Osmanthus fragrans *	Oleaceae	MG682073	MG682013	MG682033	MG682053
	CFCC 52108	China	* Osmanthus fragrans *	Oleaceae	MG682074	MG682014	MG682034	MG682054
	CFCC 52107	China	* Osmanthus fragrans *	Oleaceae	MG682075	MG682015	MG682035	MG682055
	CFCC 52109	China	* Osmanthus fragrans *	Oleaceae	MG682076	MG682016	MG682036	MG682056
* Dendrostoma parasiticum *	CFCC 52761	China	* Castanea mollissima *	Fagaceae	MH542480	MH542636	MH545429	MH545387
	CFCC 52763	China	* Castanea mollissima *	Fagaceae	MH542481	MH542637	MH545430	MH545388
	CFCC 52762*	China	* Quercus wutaishanica *	Fagaceae	MH542482	MH542638	MH545431	MH545389
	CFCC 52764	China	* Quercus aliena *	Fagaceae	MH542483	MH542639	MH545432	MH545390
	CFCC 52765	China	* Castanea mollissima *	Fagaceae	MH542484	MH542640	MH545433	MH545391
	CFCC 52766	China	Quercus aliena var. acuteserrata	Fagaceae	MH542485	MH542641	MH545434	MH545392
* Dendrostoma qinlingense *	CFCC 52732*	China	* Quercus wutaishanica *	Fagaceae	MH542471	MH542633	MH545420	MH545378
	CFCC 52733	China	Quercus aliena var. acuteserrata	Fagaceae	MH542472	MH542634	MH545421	MH545379
* Dendrostoma quercinum *	CFCC 52103*	China	* Quercus acutissima *	Fagaceae	MG682077	MG682017	MG682037	MG682057
	CFCC 52104	China	* Quercus acutissima *	Fagaceae	MG682078	MG682018	MG682038	MG682058
	CFCC 52105	China	* Quercus acutissima *	Fagaceae	MG682079	MG682019	MG682039	MG682059
* Dendrostoma quercus *	CFCC 52734	China	*Quercus* sp.	Fagaceae	MH542473	NA	MH545422	MH545380
	CFCC 52735	China	*Quercus* sp.	Fagaceae	MH542474	NA	MH545423	MH545381
	CFCC 52737	China	*Quercus* sp.	Fagaceae	MH542475	NA	MH545424	MH545382
	CFCC 52739*	China	*Quercus* sp.	Fagaceae	MH542476	MH542635	MH545425	MH545383
	CFCC 52738	China	*Quercus* sp.	Fagaceae	MH542477	NA	MH545426	MH545384
	CFCC 52736	China	*Quercus* sp.	Fagaceae	MH542478	NA	MH545427	MH545385
	CFCC 52740	China	*Quercus* sp.	Fagaceae	MH542479	NA	MH545428	MH545386
* Dendrostoma shaanxiense *	CFCC 52741*	China	* Castanea mollissima *	Fagaceae	MH542486	MH542642	MH545435	MH545393
	CFCC 52742	China	* Castanea mollissima *	Fagaceae	MH542487	MH542643	MH545436	MH545394
* Dendrostoma shandongense *	CFCC 52759*	China	* Castanea mollissima *	Fagaceae	MH542504	MH542652	MH545453	MH545411
	CFCC 52760	China	* Castanea mollissima *	Fagaceae	MH542505	MH542653	MH545454	MH545412
* Disculoides eucalypti *	CBS 132183*	Australia	*Eucalyptus* sp.	Myrtaceae	JQ685517	JQ685523	MH545455	MH545413
* Disculoides eucalyptorum *	CBS 132184*	Australia	* Eucalyptus viminalis *	Myrtaceae	JQ685518	JQ685524	MH545456	MH545414

A partition homogeneity test with heuristic search and 1000 replicates was performed using PAUP v. 4.0b10 to assess the discrepancy amongst the ITS, LSU, *TEF1-α* and *RPB2* sequence datasets in reconstructing phylogenetic trees. MP analysis was run using a heuristic search option of 1000 search replicates with random-additions of sequences with a tree bisection and reconnection algorithm. Maxtrees were set to 5000, branches of zero length were collapsed and all equally parsimonious trees were saved. Other calculated parsimony scores were tree length (TL), consistency index (CI), retention index (RI) and rescaled consistency (RC). ML analysis was performed using a GTR site substitution model including a gamma-distributed rate heterogeneity and a proportion of invariant sites (Guindon et al. 2010). The branch support was evaluated using a bootstrapping method of 1000 replicates (Hillis and Bull 1993). Phylograms were shown using FigTree v. 1.3.1 (Rambaut and Drummond 2010). Novel sequences generated in the current study were deposited in GenBank (Table 1) and the aligned matrices used for phylogenetic analyses in TreeBASE (accession number: S22929).

## Results

### Phylogenetic analyses

**Figure 2.**
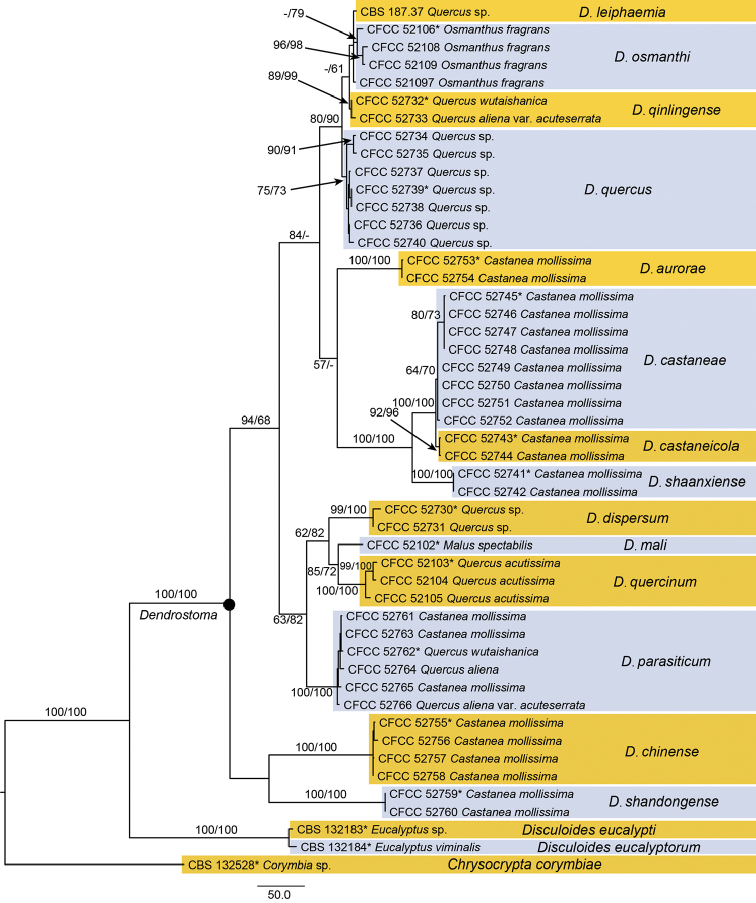
Phylogenetic tree based on an MP analysis of a combined DNA dataset of ITS, LSU, *TEF1-α* and *RPB2* gene sequences for the species of *Dendrostoma*. Bootstrap values ≥ 50% for MP and ML analyses are presented at the branches. Isolates representing ex-type material are marked with *.

The alignment based on the combined sequence dataset (ITS, LSU, *TEF1-α* and *RPB2*) included 46 ingroup taxa and three outgroup taxa, comprising 3536 characters in the aligned matrix. Of these, 2612 characters were constant, 175 variable characters were parsimony-uninformative and 749 characters were parsimony informative (101 from ITS, 21 from LSU, 389 from *TEF1-α* and 238 from *RPB2*). The MP analysis resulted in 108 equally most parsimonious trees (TL = 1590, CI = 0.744, RI = 0.897, RC = 0.668); the first tree is shown in Fig. 2. The phylogram based on the four gene sequences indicated 10 new species in *Dendrostoma* (Fig. 2), as described below.

### Taxonomy

#### Dendrostoma

Taxon classificationFungiDiaporthalesErythrogloeaceae

X.L. Fan & C.M. Tian, Persoonia 40: 126 (2018)

##### Type species.

*Dendrostomamali* X.L. Fan & C.M. Tian.

##### Description.

Sexual morph: *Pseudostromata* small to large, distinct, circular, erumpent, consisting of an inconspicuous ectostromatic disc, semi-immersed to superficial, causing a pustulate bark surface. *Ectostromatic disc* flat or concave, orange, surrounded by bark flaps. *Central column* beneath the disc more or less conical. *Stromatic zones* lacking. *Ascomata* perithecial, conspicuous, umber to fuscous black, embedded in orange to umber pseudostromatic tissue, regularly scattered, surrounding the ectostromatic disc, with small to long ostioles that emerge within the ectostromatic disc. *Ostioles* flat in the disc or sometimes slightly projecting, cylindrical, sometimes obscuring the disc, covered by an orange, umber to fuscous black crust. *Paraphyses* deliquescent. *Asci* fusoid, 8-spored, 2–3-seriate, with an apical ring, becoming detached from the perithecial wall. *Ascospores* hyaline, fusoid to cylindrical, symmetrical to asymmetrical, straight to curved, bicellular, with a median septum, constricted at the septum, smooth, multiguttulate. Asexual morph: *Conidiomata* pycnidial, spherical to conical to pulvinate, occurring separately, immersed to semi-immersed in bark; wall of several layers of yellow *textura angularis*. *Central column* beneath the disc conical or not. *Conidiophores* reduced to conidiogenous cells. *Conidiogenous cells* lining the inner walls of cavity, hyaline, smooth, subcylindrical to ampulliform. *Conidia* hyaline, aseptate, smooth, multiguttulate or not, thin-walled, ellipsoid to fusoid, straight to curved.

#### Dendrostoma aurorae

Taxon classificationFungiDiaporthalesErythrogloeaceae

C.M. Tian & N. Jiang sp. nov.

MB826795

Figure 3

##### Diagnosis.

*Dendrostomaaurorae* differs from *D.chinensis* and *D.shandongense* by the existence of obvious central column.

##### Holotype.

CHINA. Shaanxi Province: Lan’gao County, chestnut plantation, 32°13'43"N, 109°00'44"E, 1820 m a.s.l., on branches of *Castaneamollissima*, 3 Jul. 2017, N. Jiang (holotype: BJFC-S1561; ex-type culture: CFCC 52753).

##### Etymology.

*Aurorae*, referring to the orange conidiomata with exuding conidial tendrils.

##### Description.

*Sexual morph* not observed. *Asexual morph*: *Conidiomata* pycnidial, conical to pulvinate, occurring separately, bright yellow to orange, semi-immersed in bark, 300–500 μm high, 800–1400 μm diam.; wall of several layers of bright yellow *textura angularis*; conidiomata exuding slimy orange masses of conidia; *central column* beneath the disc more or less conical, pale yellow. *Conidiophores* reduced to conidiogenous cells. *Conidiogenous cells* lining the inner walls of the cavity, hyaline, smooth, subcylindrical to ampulliform, 4–15 × 2.5–4 μm. *Conidia* hyaline, aseptate, smooth, multiguttulate, thin-walled, ellipsoid to fusoid, straight to curved, (7.2–)8.1–9.8(–10.3) × (2.1–)2.3–2.6(–2.8) μm, l/w = (2.7–)3.2–4.1(–4.2) (n = 50).

**Figure 3.**
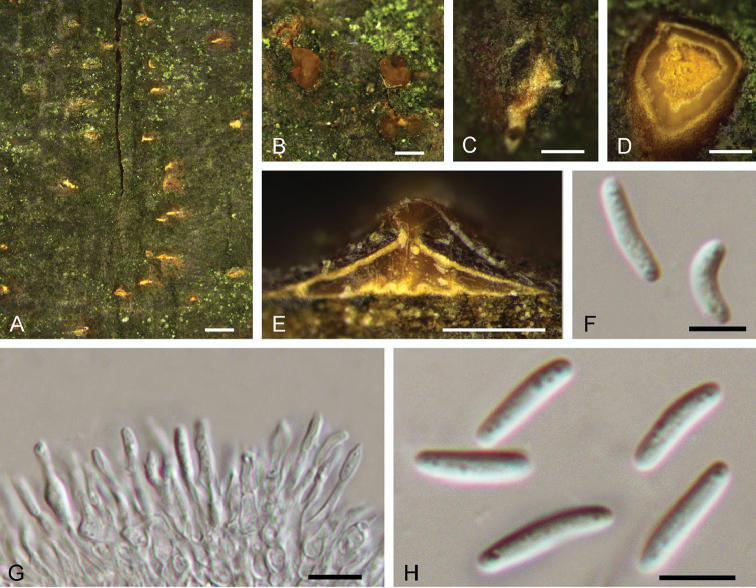
Morphology of *Dendrostomaaurorae* from *Castaneamollissima* (BJFC-S1561). **A–C** Habit of conidiomata on branches **D** Transverse section of conidioma **E** Longitudinal section through conidioma **F, H** Conidia **G** Conidiogenous cells. Scale bars: 1 mm (**A**); 0.5 mm (**B, C, E**); 0.2 mm (**D**); 5 μm (**F, H**); 10 μm (**G**).

##### Culture characters.

On PDA, cultures are initially white, becoming isabelline after 2 weeks. The colonies are flat with irregular edge; texture uniform within 1 month at 25 °C in the dark.

##### Additional specimen examined.

CHINA. Shaanxi Province: Lan’gao County, chestnut plantation, 32°13'43"N, 109°00'44"E, 1820 m a.s.l., on branches of *Castaneamollissima*, 3 Jul. 2017, N. Jiang, living culture CFCC 52754 (BJFC-S1562).

##### Notes.

*Dendrostomaaurorae* was discovered on stems of dying chestnut trees and appears morphologically similar to the chestnut blight pathogen, *Cryphonectriaparasitica*. However, these two diaporthalean pathogens can be distinguished by the existence of a central column inside the conidiomata of *Dendrostomaaurorae*. In the genus *Dendrostoma*, *D.aurorae* differs from *D.chinensis* and *D.shandongense* by the existence of an obvious central column.

#### Dendrostoma castaneae

Taxon classificationFungiDiaporthalesErythrogloeaceae

C.M. Tian & N. Jiang sp. nov.

MB826796

Figure 4

##### Diagnosis.

*Dendrostomacastaneae* is distinguished from the phylogenetically closely related species *D.castaneicola* by its narrower conidia.

##### Holotype.

CHINA. Hebei Province: Xinglong County, chestnut plantation, 40°21'44"N, 117°51'29"E, 256 m a.s.l., on branches of *Castaneamollissima*, 27 Apr. 2018, N. Jiang & C.M. Tian (holotype: BJFC-S1553; ex-type culture: CFCC 52745).

##### Etymology.

*Castaneae*, referring to the host genus, *Castanea*.

##### Description.

*Sexual morph* not observed. *Asexual morph*: *Conidiomata* pycnidial, pulvinate, occurring separately, bright yellow to orange, immersed in bark, 400–600 μm high, 900–2200 μm diam.; wall of several layers of brown *textura angularis*; *central column* beneath the disc irregular, pale yellow. *Conidiophores* reduced to conidiogenous cells. *Conidiogenous cells* lining the inner walls of the cavity, hyaline, smooth, subcylindrical to ampulliform, 3–10 × 2–3.5 μm. *Conidia* hyaline, aseptate, smooth, multiguttulate, thin-walled, ellipsoid, straight to curved, (9.3–)10.4–12.3(–13.3) × (2.1–)2.2–2.7(–2.9) μm, l/w = (3.4–)4.2–5.2(–5.9) (n = 50).

**Figure 4.**
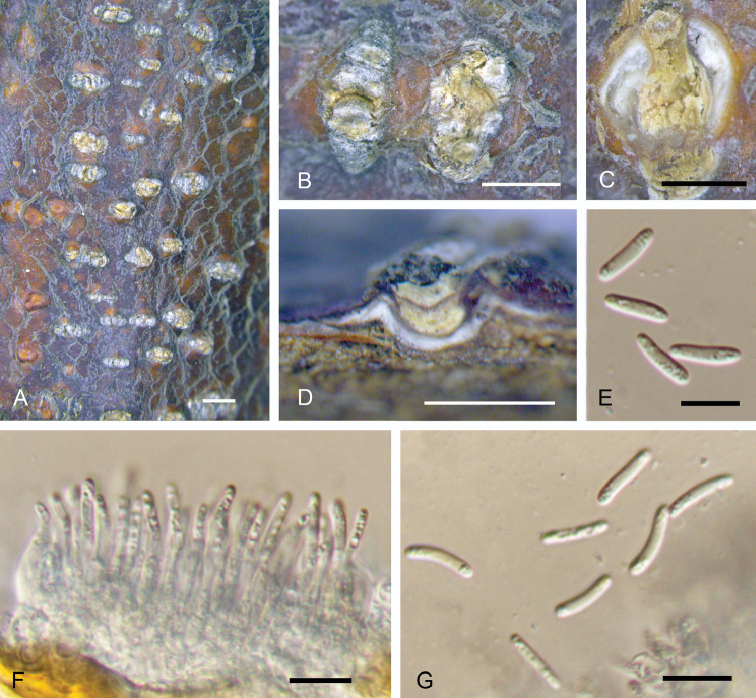
Morphology of *Dendrostomacastaneae* from *Castaneamollissima* (BJFC-S1553). **A, B** Habit of conidiomata on branches **C** Transverse section of conidioma **D** Longitudinal section through conidioma **E, G** Conidia **F** Conidiogenous cells. Scale bars: 1 mm (**A–D**); 10 μm (**E–G**).

##### Culture characters.

On PDA, cultures are initially white, exhibiting grey after 2 weeks. Colonies are flat with irregular edge; texture initially uniform, producing concentric circles with faint orange conidiomata distributed outside the rim within 1 month at 25 °C in the dark.

##### Additional specimens examined.

CHINA. Hebei Province: Chengde City, Xinglong County, chestnut plantation, 40°21'44"N, 117°51'29"E, 256 m a.s.l., on branches of *Castaneamollissima*, 27 Apr. 2018, N. Jiang & C.M. Tian, living culture CFCC 52748 (BJFC-S1556); Hebei Province: Chengde City, Xinglong County, chestnut plantation, 40°21'44"N, 117°51'29"E, 256 m a.s.l., on branches of *Castaneamollissima*, 27 Apr. 2018, N. Jiang & C.M. Tian, living culture CFCC 52751 (BJFC-S1557); Hebei Province: Chengde City, Xinglong County, chestnut plantation, 40°21'44"N, 117°51﻿﻿'29"E, 256 m a.s.l., on branches of *Castaneamollissima*, 27 Apr. 2018, N. Jiang & C.M. Tian, living culture CFCC 52747 (BJFC-S1559); Hebei Province: Chengde City, chestnut plantation, 40°37'39"N, 118°27'22"E, 256 m a.s.l., on branches of *Castaneamollissima*, 28 Apr. 2018, N. Jiang & C.M. Tian, living culture CFCC 52750 (BJFC-S1558); Hebei Province: Chengde City, chestnut plantation, 40°37'39"N, 118°27'22"E, 256 m a.s.l., on branches of *Castaneamollissima*, 28 Apr. 2018, N. Jiang & C.M. Tian, living culture CFCC 52752 (BJFC-S1560); Tianjin City: Jizhou District, chestnut plantation, 40°06'33"N, 117°42'45"E, 185 m a.s.l., on branches of *Castaneamollissima*, 25 Apr. 2018, N. Jiang & C.M. Tian, living culture CFCC 52749 (BJFC-S1554); Tianjin City: Jizhou District, chestnut plantation, 40°06'33"N, 117°42'45"E, 185 m a.s.l., on branches of *Castaneamollissima*, 25 Apr. 2018, N. Jiang & C.M. Tian, living culture CFCC 52746 (BJFC-S1555).

##### Notes.

*Dendrostomacastaneae* is the most common species in this genus occurring on the host *Castaneamollissima* in China and is associated with canker symptoms on stems and branches. As shown in Fig. 2, *Dendrostomacastaneae* is the closest relative of *D.castaneicola*; however, they can be distinguished by conidial width (2.2–2.7 μm in *D.castaneae* vs. 3.2–3.8 μm in *D.castaneicola*).

#### Dendrostoma castaneicola

Taxon classificationFungiDiaporthalesErythrogloeaceae

C.M. Tian & N. Jiang sp. nov.

MB826797

Figure 5

##### Diagnosis.

*Dendrostomacastaneicola* differs from the two phylogenetically closely related species, *D.castaneae* and *D.shaanxiense*, by its white central column.

##### Holotype.

CHINA. Hebei Province: Chengde City, chestnut plantation, 40°24'32"N, 117°28'55"E, 262 m a.s.l., on branches of *Castaneamollissima*, 28 Apr. 2018, N. Jiang & C.M. Tian (holotype: BJFC-S1551; ex-type culture: CFCC 52743).

##### Etymology.

*Castaneicola*, referring to the host genus, *Castanea*.

##### Description.

*Sexual morph* not observed. *Asexual morph*: *Conidiomata* pycnidial, conical to pulvinate, occurring separately, reddish-orange, semi-immersed in bark, 300–550 μm high, 900–1600 μm diam.; wall of several layers of faint yellow *textura angularis*; *central column* beneath the disc more or less conical, white. *Conidiophores* reduced to conidiogenous cells. *Conidiogenous cells* lining the inner walls of the cavity, hyaline, smooth, subcylindrical to ampulliform, 5–14 × 2–3.5 μm. *Conidia* hyaline, aseptate, smooth, multiguttulate, thin-walled, ellipsoid to fusoid, straight, (9.3–)10.5–12.8(–13.8) × (3.1–)3.2–3.8(–4.1) μm, l/w = (2.3–)3–4(–4.4) (n = 50).

**Figure 5.**
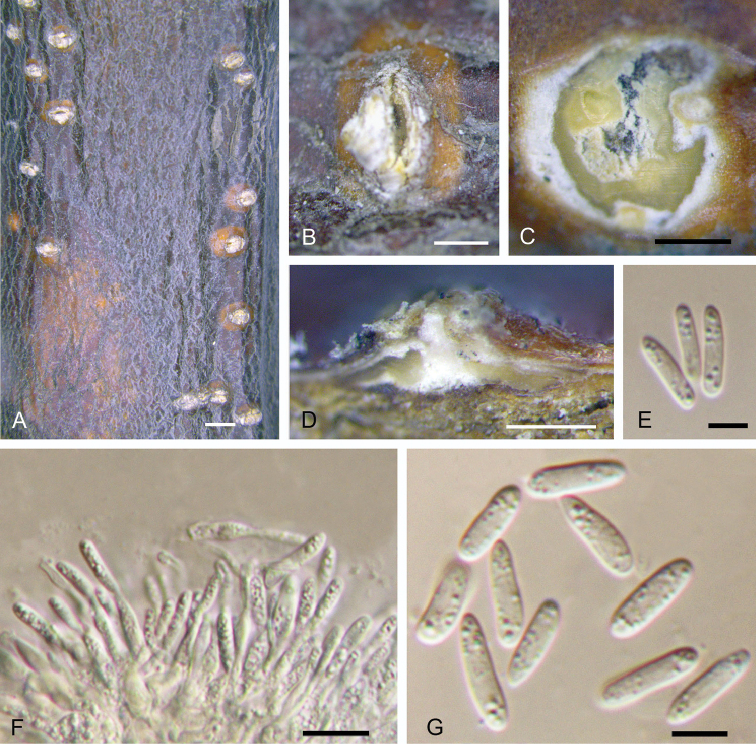
Morphology of *Dendrostomacastaneicola* from *Castaneamollissima* (BJFC-S1551). **A, B** Habit of conidiomata on branches **C** Transverse section of conidioma **D** Longitudinal section through conidioma **E, G** Conidia **F** Conidiogenous cells. Scale bars: 1 mm (**A**); 0.5 mm (**B–D**); 5 μm (**E, G**); 10 μm (**F**).

##### Culture characters.

On PDA, cultures are initially white, becoming black after 2 weeks. The colonies are flat with irregular edge; texture uniform, producing a circle with faint orange conidiomata distributed along the edge of the circle within 1 month at 25 °C in the dark.

##### Additional specimen examined.

CHINA. Hebei Province: Chengde City, Xinglong County, chestnut plantation, 40°21'44"N, 117°51'29"E, 256 m a.s.l., on branches of *Castaneamollissima*, 27 Apr. 2018, N. Jiang & C.M. Tian, living culture CFCC 52744 (BJFC-S1552).

##### Notes.

*Dendrostomacastaneicola*, *D.castaneae* and *D.shaanxiense* comprise three closely related pathogen species causing chestnut canker diseases in China, all three species occurring on *Castaneamollissima*. They differ with regard to conidiomatal characteristics, including conidial dimensions (Table 2) and the central column colour (pale yellow central column in *D.castaneae* vs. white in *D.castaneicola* vs. bright yellow in *D.shaanxiense*). Additionally, *Dendrostomashaanxiense* was only discovered in the Shaanxi Province, whereas *D.castaneae* and *D.castaneicola* were both distributed in Hebei Province.

**Table 2.** Conidial size of *Dendrostoma* species from natural host barks, species with * were measured from conidia produced in PDA.

**Species**	**Conidial length (μm)**	**Conidial width (μm)**	**Length/width ratio**
* Dendrostoma aurorae *	8.1–9.8	2.3–2.6	3.2–4.1
* Dendrostoma castaneae *	10.4–12.3	2.2–2.7	4.2–5.2
* Dendrostoma castaneicola *	10.5–12.8	3.2–3.8	3–4
* Dendrostoma chinense *	7.7–9.1	3.4–3.7	2.2–2.6
* Dendrostoma dispersum *	11.1–12.2	2–2.3	4.9–5.9
Dendrostomamali*	3.5–4.5	2–2.5	NA
Dendrostomaosmanthi*	7.5–10.5	2–2.5	NA
* Dendrostoma parasiticum *	9.3–11.7	2.8–3.3	3–3.9
* Dendrostoma qinlingense *	16–18	3.3–3.7	4.4–5.2
Dendrostomaquercinum*	10.5–14	2.5	NA
* Dendrostoma quercus *	13.3–16.1	3.5–4.2	3.3–4.4
* Dendrostoma shaanxiense *	9.5–11.1	2.5–3.1	3.3–4.2
* Dendrostoma shandongense *	8.1–8.8	3.8–4.3	1.9–2.3

#### Dendrostoma chinense

Taxon classificationFungiDiaporthalesErythrogloeaceae

C.M. Tian & N. Jiang sp. nov.

MB826798

Figure 6

##### Diagnosis.

*Dendrostomachinense* differs from *D.shandongense* by the appearance of conidiomata and is again similar to *D.shandongense* in its conidial characteristics.

##### Holotype.

CHINA. Shandong Province: Rizhao City, Donggang District, chestnut plantation, 35°42'28"N, 119°46'23"E, 452 m a.s.l., on branches of *Castaneamollissima*, 14 Apr. 2017, N. Jiang (holotype: BJFC-S1563; ex-type culture: CFCC 52755).

##### Etymology.

*Chinense*, referring to the country, China.

##### Description.

*Sexual morph* not observed. *Asexual morph*: *Conidiomata* pycnidial, spherical, occurring separately, black, semi-immersed in bark, 250–450 μm high, 600–850 μm diam.; wall of several layers of white *textura angularis*. *Conidiophores* reduced to conidiogenous cells. *Conidiogenous cells* lining the inner walls of the cavity, hyaline, smooth, ampulliform, 7–14 × 1–2.5 μm. *Conidia* hyaline, aseptate, smooth, multiguttulate or not, thin-walled, fusoid to ellipsoid, apex acutely rounded, base truncate, (6.9–)7.7–9.1(–9.7) × (3.3–)3.4–3.7(–3.9) μm, l/w = (1.9–)2.2–2.6(–2.7) (n = 50).

**Figure 6.**
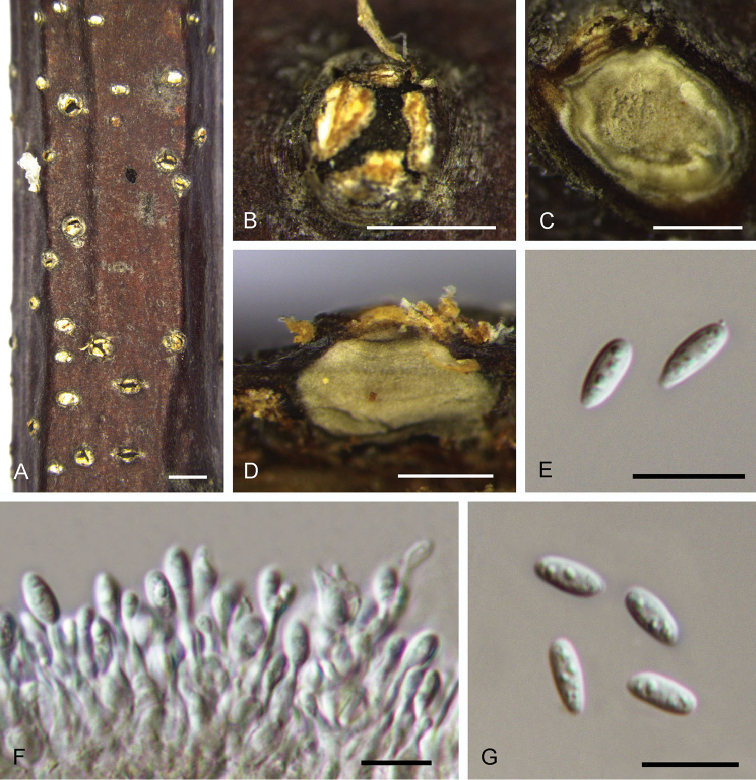
Morphology of *Dendrostomachinense* from *Castaneamollissima* (BJFC-S1563). **A, B** Habit of conidiomata on branches **C** Transverse section of conidioma **D** Longitudinal section through conidioma **E, G** Conidia **F** Conidiogenous cells. Scale bars: 1 mm (**A**); 0.5 mm (**B–D**); 10 μm (**E–G**).

##### Culture characters.

On PDA, cultures are initially white, becoming olive green in the outer zone after 2 weeks. Colonies are flat with a regular edge; texture uniform within 1 month at 25 °C in the dark.

##### Additional specimens examined.

CHINA. Shandong Province: Rizhao City, Donggang District, chestnut plantation, 35°42'28"N, 119°46'23"E, 452 m a.s.l., on branches of *Castaneamollissima*, 14 Apr. 2017, N. Jiang, living culture CFCC 52756 (BJFC-S1564); Hebei Province: Chengde City, chestnut plantation, 40°24'32"N, 117°28'55"E, 262 m a.s.l., on branches of *Castaneamollissima*, 29 Apr. 2018, N. Jiang & C.M. Tian, living culture CFCC 52757 (BJFC-S1565); Hebei Province: Chengde City, chestnut plantation, 40°24'32"N, 117°28'55"E, 262 m a.s.l., on branches of *Castaneamollissima*, 29 Apr. 2018, N. Jiang & C.M. Tian, living culture CFCC 52757 (BJFC-S1566).

##### Notes.

*Dendrostomachinense* and *D.shandongense* have been occasionally discovered on the same branches and share similar conidial shape and dimensions. However, the conidiomatal appearance of these two species is quite different (black conidiomata in *Dendrostomachinense* vs. orange conidiomata in *D.shandongense*).

#### Dendrostoma dispersum

Taxon classificationFungiDiaporthalesErythrogloeaceae

C.M. Tian & N. Jiang sp. nov.

MB826799

Figure 7

##### Diagnosis.

*Dendrostomadispersum* can be distinguished from the phylogenetically closely related *D.mali* and *D.quercinum* based on its conidial dimensions.

##### Holotype.

CHINA. Shaanxi Province: Beijing City: Mentougou District, Xiaolongmen Forest Park, 39°55'52"N, 115°45'15"E, 1670 m a.s.l., on branches of *Quercus* sp., 15 Aug. 2017, N. Jiang & X.L. Fan (holotype: BJFC-S1537; ex-type culture: CFCC 52730).

##### Etymology.

*Dispersum*, referring to the conidiomata scattered on the bark surface.

##### Description.

*Sexual morph* not observed. *Asexual morph*: *Conidiomata* pycnidial, conical to spherical, occurring separately, bright yellow, semi-immersed in bark, 500–800 μm high, 900–1500 μm diam.; wall of several layers of bright yellow *textura angularis*; *central column* beneath the disc conical, bright yellow. *Conidiophores* reduced to conidiogenous cells. *Conidiogenous cells* lining the inner walls of the cavity, hyaline, smooth, subcylindrical to ampulliform, 6–15 × 2.5–5 μm. *Conidia* hyaline, aseptate, smooth, multiguttulate, thin-walled, ellipsoid to fusoid, straight to curved, (10.9–)11.1–12.2(–12.8) × (1.9–)2–2.3(–2.4) μm, l/w = (4.8–)4.9–5.9(–6.3) (n = 50).

**Figure 7.**
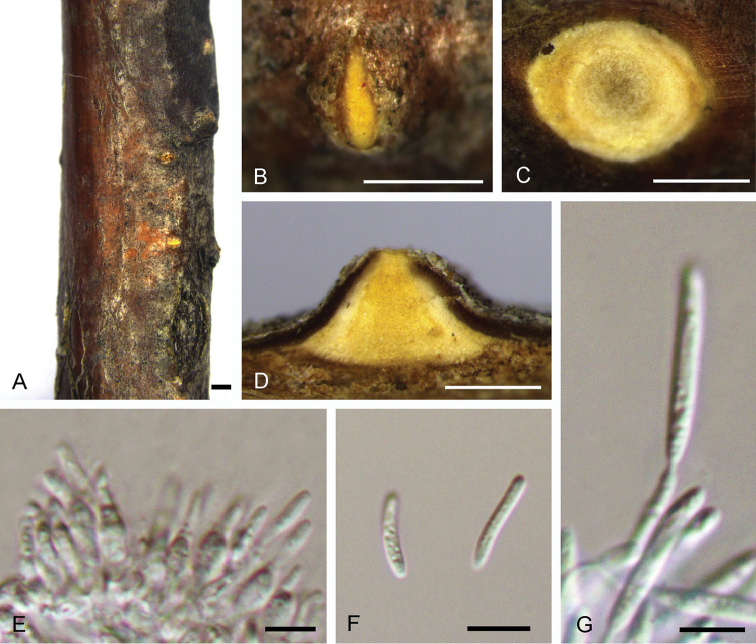
Morphology of *Dendrostomadispersum* from *Quercus* sp. (BJFC-S1537). **A, B** Habit of conidiomata on branches **C** Transverse section of conidioma **D** Longitudinal section through conidioma **E, G** Conidiogenous cells **F** Conidia. Scale bars: 1 mm (**A**); 0.5 mm (**B–D**); 10 μm (**E, F**), 5 μm (**G**).

##### Culture characters.

On PDA, cultures are initially white, becoming faint yellow after 2 weeks. The colonies are flat with regular edge; texture uniform, producing concentric circles within 1 month at 25 °C in the dark.

##### Additional specimen examined.

CHINA. Beijing City: Yanqing District, Yudu Mountain, 40°53'48"N, 115°54'48"E, 840 m a.s.l., on branches of *Quercus* sp., 12 Mar. 2018, N. Jiang, X.L. Fan, Y.M. Liang & C.M. Tian, living culture CFCC 52731 (BJFC-S1538).

##### Notes.

*Dendrostomadispersum* is phylogenetically close to *D.mali* and *D.quercinum* (Fig. 2). Conidial dimensions of *Dendrostomamali* and *D.quercinum* were described from PDA plates (Fan et al. 2018) and *D.dispersum* can be differentiated from *D.mali* by having much longer conidia (11.1–12.2 μm in *D.dispersum* vs. 3–4.5 μm in *D.mali*) and from *D.quercinum* by narrower conidia (2–2.3 μm in *D.dispersum* vs. 2.5–3 μm in *D.quercinum*).

#### Dendrostoma parasiticum

Taxon classificationFungiDiaporthalesErythrogloeaceae

C.M. Tian & N. Jiang sp. nov.

MB826822

Figure 8

##### Diagnosis.

*Dendrostomaparasiticum* is distinguished from *D.quercus* by its shorter and narrower conidia.

##### Holotype.

CHINA. Shaanxi Province: Shangluo City, Zhashui County, Longtougou Village, 33°39'27"N, 109°07'15"E, 2504 m a.s.l., on branches of *Quercuswutaishanica*, 8 Jul. 2017, N. Jiang (holotype: BJFC-S1570; ex-type culture: CFCC 52762).

##### Etymology.

*Parasiticum*, referring to the fungus causing canker diseases on different hosts.

##### Description.

*Sexual morph* not observed. *Asexual morph*: *Conidiomata* pycnidial, conical to spherical, occurring separately, yellow, semi-immersed in bark, 350–600 μm high, 1000–1800 μm diam.; wall of several layers of bright yellow *textura angularis*; *central column* beneath the disc conical, bright yellow. *Conidiophores* reduced to conidiogenous cells. *Conidiogenous cells* lining the inner walls of the cavity, hyaline, smooth, subcylindrical to ampulliform, 7–12 × 2–3.5 μm. *Conidia* hyaline, aseptate, smooth, multiguttulate, thin-walled, fusoid, straight, (9.2–)9.3–11.7(–13.6) × (2.7–)2.8–3.3(–3.6) μm, l/w = (2.7–)3–3.9(–4.2) (n = 50).

**Figure 8.**
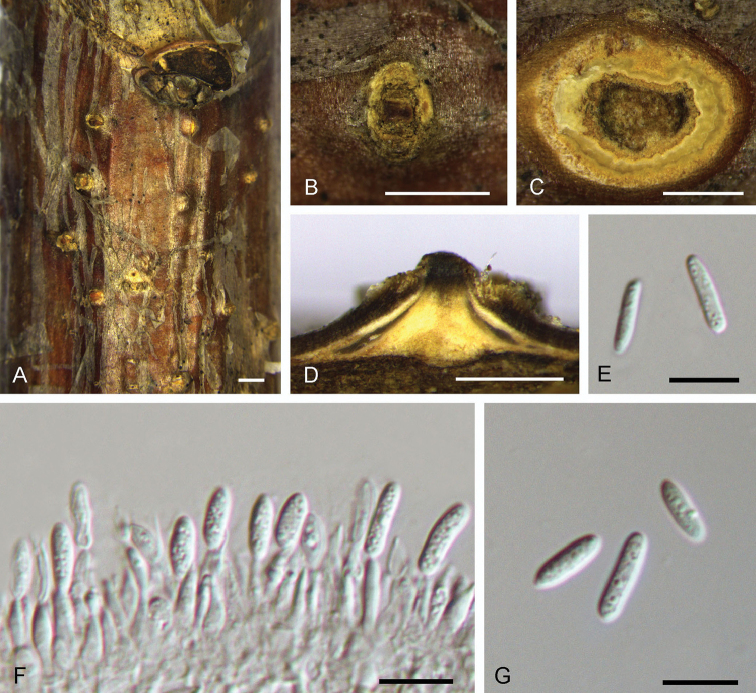
Morphology of *Dendrostomaparasiticum* from *Quercuswutaishanica* (BJFC-S1570). **A, B** Habit of conidiomata on branches **C** Transverse section of conidioma **D** Longitudinal section through conidioma **E, G** Conidia **F** Conidiogenous cells. Scale bars: 2 mm (**A**); 1 mm (**B**); 0.5 mm (**C, D**); 10 μm (**E–G**).

##### Culture characters.

On PDA, cultures are initially white, becoming dark orange after 2 weeks. The colonies are flat with irregular edge; texture uniform, producing concentric circles within 1 month at 25 °C in the dark.

##### Additional specimens examined.

CHINA. Shaanxi Province: Shangluo City, Zhashui County, chestnut plantation, 33°39'27"N, 109°07'15"E, 2504 m a.s.l., on branches of *Castaneamollissima*, 8 Jul. 2017, N. Jiang, living culture CFCC 52762 (BJFC-S1569); Shaanxi Province: Ankang City, Xiangxidong Park, 32°40'32"N, 109°18'57"E, 2504 m a.s.l., on branches of *Castaneamollissima*, 29 Jun. 2017, N. Jiang, living culture CFCC 52763 (BJFC-S1571); Beijing City: Mentougou District, Xiaolongmen Forest Park, 39°17'25"N, 115°45'23"E, 452 m a.s.l., on branches of *Castaneamollissima*, 17 Aug. 2017, N. Jiang & X.L. Fan, living culture CFCC 52764 (BJFC-S1572); Beijing City: Yanqing District, Yudu Mountain, 40°53'48"N, 115°54'48"E, 840 m a.s.l., on branches of *Quercusaliena*, 12 Mar. 2017, N. Jiang, X.L. Fan, Y.M. Liang & C.M. Tian, living culture CFCC 52765 (BJFC-S1573); Hebei Province: Chengde City, chestnut plantation, 40°24'32"N, 117°28'55"E, 262 m a.s.l., on branches of Quercusalienavar.acutiserrata, 15 Oct. 2017, N. Jiang, living culture CFCC 52766 (BJFC-S1574).

##### Notes.

*Dendrostomaparasiticum* constitutes a widely distributed species occurring on several Fagaceae tree species including *Castaneamollissima*, *Quercusaliena*, Q.alienavar.acuteserrata and *Q.wutaishansea*. *Dendrostomaparasiticum* appears to be associated with tree dieback, canker and even tree death, although its pathogenicity remains unproven. *Dendrostomaparasiticum* is close to *D.quercus* in the phylogram (Fig. 2), but differs from *D.quercus* with shorter (9.3–11.7 μm in *D.parasiticum* vs. 13.3–16.1 μm in *D.quercus*) and narrower (2.8–3.3 μm in *D.parasiticum* vs. 3.5–4.2 μm in *D.quercus*) conidia.

#### Dendrostoma qinlingense

Taxon classificationFungiDiaporthalesErythrogloeaceae

C.M. Tian & N. Jiang sp. nov.

MB826823

Figure 9

##### Diagnosis.

*Dendrostomaqinlingense* produces the largest conidia amongst known species of the genus.

##### Holotype.

CHINA. Baoji City, Mei County, Taibai Mountain, 34°15'43"N, 107°88'42"E, 2752 m a.s.l., on branches of *Quercuswutaishanica*, 13 Jul. 2017, N. Jiang (holotype: BJFC-S1539; ex-type culture: CFCC 52732).

##### Etymology.

*Qinlingense*, referring to the Qinling Mountain.

##### Description.

*Sexual morph* not observed. *Asexual morph*: *Conidiomata* pycnidial, conical to pulvinate, occurring separately, dark yellow, semi-immersed in bark, 400–700 μm high, 1100–1600 μm diam.; wall of several layers of bright yellow *textura angularis*; *central column* beneath the disc conical, dark orange. *Conidiophores* reduced to conidiogenous cells. *Conidiogenous cells* lining the inner walls of the cavity, hyaline, smooth, ampulliform, 6–22 × 2–3.5 μm. *Conidia* hyaline, aseptate, smooth, multiguttulate, thin-walled, fusoid, straight, (15.6–)16–18(–18.6) × (3.1–)3.3–3.7(–3.8) μm, l/w = (4.2–)4.4–5.2(–5.8) (n = 50).

**Figure 9.**
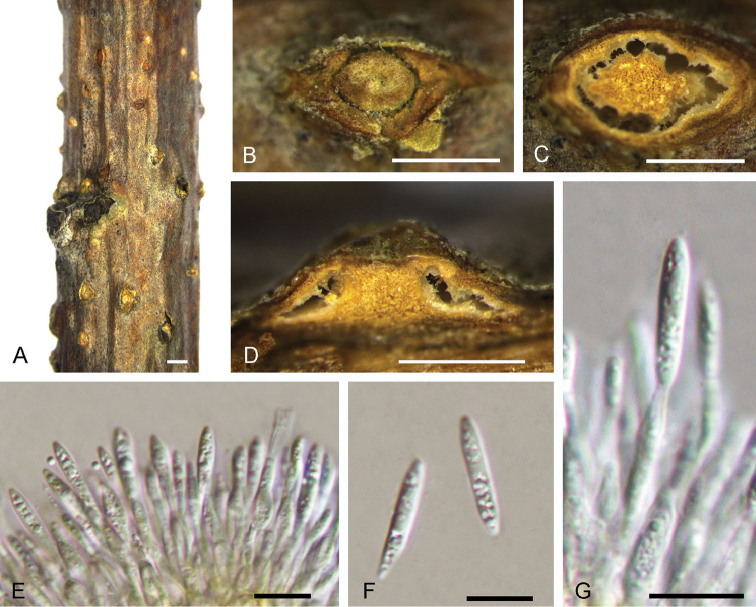
Morphology of *Dendrostomaqinlingense* from *Quercuswutaishanica* (BJFC-S1539). **A, B** Habit of conidiomata on branches **C** Transverse section of conidioma **D** Longitudinal section through conidioma **E, G** Conidiogenous cells **F** Conidia. Scale bars: 1 mm (**A**); 0.5 mm (**B–D**); 10 μm (**E–G**).

##### Culture characters.

On PDA, cultures are initially white, exhibiting light grey after 2 weeks. The colonies are flat with irregular edge; texture uniform, producing concentric circles with sparse conidiomata irregularly distributed on the centre of the plate within 1 month at 25 °C in the dark.

##### Additional specimen examined.

CHINA. Shaanxi Province: Baoji City, Mei County, Taibai Mountain, 34°15'43"N, 107°88'42"E, 2752 m a.s.l., on branches of Quercusalienavar.acutiserrata, 13 Jul. 2017, N. Jiang, living culture CFCC 52733 (BJFC-S1540).

##### Notes.

*Dendrostomaqinlingense* was discovered on two *Quercus* species on the Qinling Mountain in northwest China. This species is phylogenetically related to *Dendrostomaosmanthi* on *Osmanthusfragrans*. However, *Dendrostomaqinlingense* differs from *D.osmanthi* by much larger conidia (16–18 × 3.3–3.7 μm in *D.qinlingense* vs. 7.5–10 × 2–2.5 μm in *D.osmanthi*).

#### Dendrostoma quercus

Taxon classificationFungiDiaporthalesErythrogloeaceae

C.M. Tian & N. Jiang sp. nov.

MB826824

Figure 10

##### Diagnosis.

*Dendrostomaquercus* is recognised by the existence of dimorphic conidia, which is unique in the genus.

##### Holotype.

CHINA. Hebei Province: Qinhuangdao City, Zu Mountain, 40°14'13"N, 119°43'28"E, 1125 m a.s.l., on branches of *Quercus* sp., 2 May 2018, N. Jiang & C.M. Tian (holotype: BJFC-S1547; ex-type culture: CFCC 52739).

##### Etymology.

*Quercus*, referring to the host genus, *Quercus*.

##### Description.

*Sexual morph*: *Pseudostromata* erumpent, consisting of an inconspicuous ectostromatic disc, semi-immersed to superficial, causing a pustulate bark surface, 1000–1500 µm diam. *Ectostromatic disc* flat or concave, pale brown to brown, sometimes concealed by ostioles, surrounded by bark flaps, 400–800 µm diam.; *central column* yellowish to brownish. *Stromatic zones* lacking. *Perithecia* conspicuous, umber to fuscous black, 350–500 µm diam. *Ostioles* 5–8 per disc, flat in the disc or sometimes slightly projecting, cylindrical, covered by an orange, umber to fuscous black crust, 60–80 µm diam. *Paraphyses* slightly deliquescent. *Asci* fusoid to slightly fusiform, 8-spored, ascospores regularly disposed, with an apical ring, 55–65 × 8–11 µm. *Ascospores* hyaline, fusoid to cylindrical, smooth, often containing one guttule per cell to multiguttulate, symmetrical to asymmetrical, straight curved, bicellular, (13.4–)13.8–15.6(–16.6) × (5.1–)5.3–5.8(–5.9) μm, l/w = (2.4–)2.5–2.8(–2.9) (n = 50). *Asexual morph*: *Conidiomata* pycnidial, conical, occurring separately, pale yellow, semi-immersed in bark, 700–1000 μm high, 700–950 μm diam.; wall of several layers of pale yellow *textura angularis*; *central column* beneath the disc conical, yellow. *Conidiophores* reduced to conidiogenous cells. *Conidiogenous cells* lining the inner walls of the cavity, hyaline, smooth, subcylindrical to ampulliform, 4.5–9 × 2–4 μm. *Conidia* hyaline, aseptate, smooth, multiguttulate, thin-walled, dimorphic, type one (> 99%) ellipsoid to fusoid, straight to curved, (11–)13.3–16.1(–16.9) × (3.4–)3.5–4.2(–4.5) μm, l/w = (2.6–)3.3–4.4(–4.9) (n = 50); type two (< 1%) fusoid, apex acutely rounded, 13–16 × 4–6 μm.

**Figure 10.**
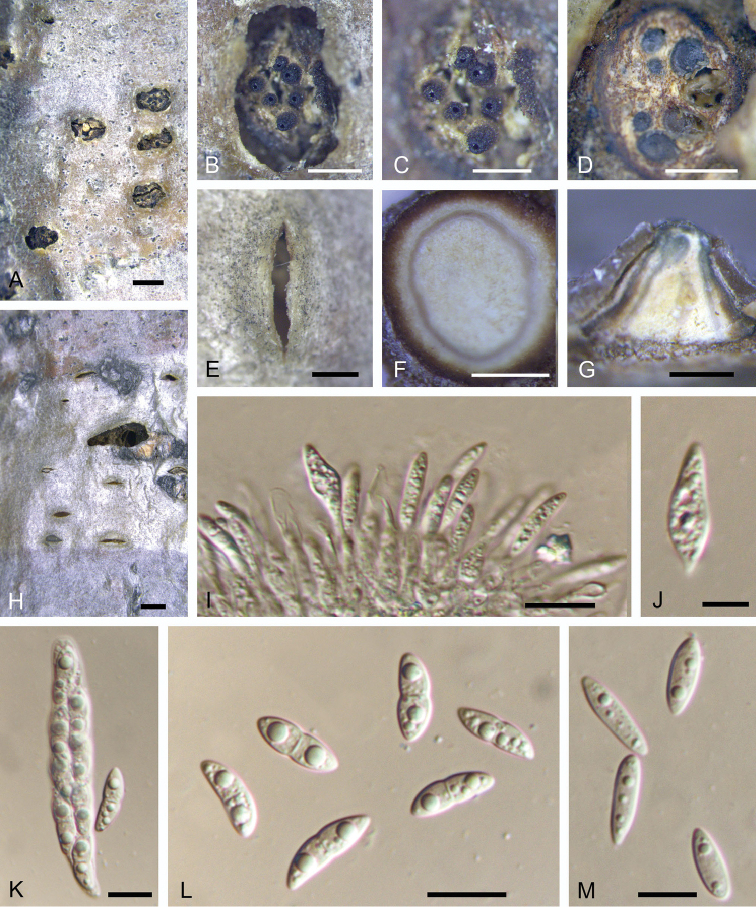
Morphology of *Dendrostomaquercus* from *Quercus* sp. (BJFC-S1547). **A–C** Habit of psedostromata on branches **D** Transverse section of pseudostroma **E, H** Habit of conidiomata on branches **F** Transverse section of conidioma **G** Longitudinal section through conidioma **I** Conidiogenous cells producing dimorphic conidia **J** Secondary conidia **K** Asci and ascospores **L** Ascospores **M** Primary conidia. Scale bars: 1 mm (**A, H**); 0.5 mm (**B–G**); 10 μm (**I, K–M**); 5 μm (**J**).

##### Culture characters.

On PDA, cultures are initially white, becoming dark grey after 2 weeks. The colonies are flat with irregular edge; texture uniform, producing concentric circles with sparse conidiomata irregularly distributed within 1 month at 25 °C in the dark.

##### Additional specimens examined.

CHINA. Hebei Province: Qinhuangdao City, Zu Mountain, 40°14'13"N, 119°43'28"E, 1125 m a.s.l., on branches of *Quercus* sp., 2 May 2018, N. Jiang & C.M. Tian, living culture CFCC 52734 (BJFC-S1548); Hebei Province: Qinhuangdao City, Zu Mountain, 40°14'13"N, 119°43'28"E, 1125 m a.s.l., on branches of *Quercus* sp., 2 May 2018, N. Jiang & C.M. Tian, living culture CFCC 52735 (BJFC-S1541); Hebei Province: Qinhuangdao City, Zu Mountain, 40°14'13"N, 119°43'28"E, 1125 m a.s.l., on branches of *Quercus* sp., 2 May 2018, N. Jiang & C.M. Tian, living culture CFCC 52736 (BJFC-S1542); Hebei Province: Qinhuangdao City, Zu Mountain, 40°14'13"N, 119°43'28"E, 1125 m a.s.l., on branches of *Quercus* sp., 2 May 2018, N. Jiang & C.M. Tian, living culture CFCC 52737 (BJFC-S1543); Hebei Province: Qinhuangdao City, Zu Mountain, 40°14'13"N, 119°43'28"E, 1125 m a.s.l., on branches of *Quercus* sp., 2 May 2018, N. Jiang & C.M. Tian, living culture CFCC 52738 (BJFC-S1544); Hebei Province: Qinhuangdao City, Zu Mountain, 40°14'13"N, 119°43'28"E, 1125 m a.s.l., on branches of *Quercus* sp., 2 May 2018, N. Jiang & C.M. Tian, living culture CFCC 52740 (BJFC-S1545).

##### Notes.

*Dendrostomaquercus* is associated with oak branch cankers and forms both sexual and asexual fruiting structures beneath cankered bark. Within the genus, *D.quercus* produces the second largest conidia, smaller only than those of *D.qinlingense* (Table 2). The presence of dimorphic conidia in *Dendrostoma*, however, is a feature unique to *D.quercus*.

#### Dendrostoma shaanxiense

Taxon classificationFungiDiaporthalesErythrogloeaceae

C.M. Tian & N. Jiang sp. nov.

MB826825

Figure 11

##### Diagnosis.

*Dendrostomashaanxiense* is distinguished from the closely related species *D.castaneae* by smaller l/w ratio and from *D.castaneicola* by its narrower conidia.

##### Holotype.

CHINA. Shaanxi Province: Ankang City, Xiangxidong Park, 32°40'32"N, 109°18'57"E, 1079 m a.s.l., on branches of *Castaneamollissima*, 1 Jul. 2017, N. Jiang (holotype: BJFC-S1549; ex-type culture: CFCC 52741).

##### Etymology.

*Shaanxiense*, referring to the Shaanxi Province in China.

##### Description.

*Sexual morph* not observed. *Asexual morph*: *Conidiomata* pycnidial, conical to pulvinate, occurring separately, dark orange, semi-immersed in bark, 350–650 μm high, 1050–1400 μm diam.; wall of several layers of bright yellow *textura angularis*; *central column* beneath the disc conical, bright yellow. *Conidiophores* reduced to conidiogenous cells. *Conidiogenous cells* lining the inner walls of the cavity, hyaline, smooth, subcylindrical to ampulliform, 5–11 × 2.5–3.5 μm. *Conidia* hyaline, aseptate, smooth, multiguttulate, thin-walled, ellipsoid to fusoid, straight to curved, (8.6–)9.5–11.1(–11.7) × (2.3–)2.5–3.1(–3.4) μm, l/w = (2.8–)3.3–4.2(–4.9) (n = 50).

**Figure 11.**
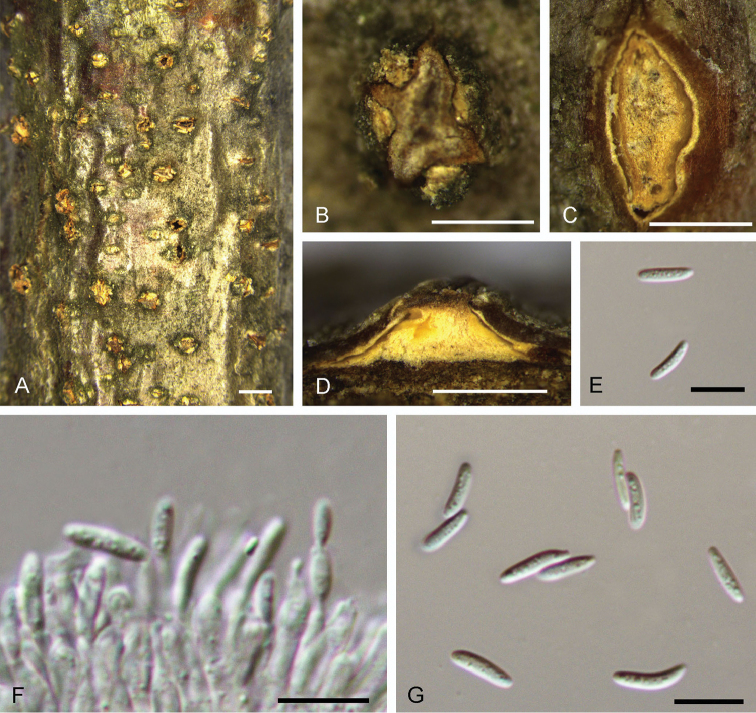
Morphology of *Dendrostomashaanxiense* from *Castaneamollissima* (BJFC-S1549). **A, B&nbsp**;Habit of conidiomata on branches **C** Transverse section of conidioma **D** Longitudinal section through conidioma **E, G** Conidia **F** Conidiogenous cells. Scale bars: 1 mm (**A**); 0.5 mm (**B–D**); 10 μm (**E–G**).

##### Culture characters.

On PDA, cultures are initially white, turning purple after 2 weeks on PDA. The colonies are flat with irregular edge; texture uniform, producing concentric circles within 1 month at 25 °C in the dark.

##### Additional specimen examined.

Shaanxi Province: Ankang City, Xiangxidong Park, 32°40'32"N, 109°18'57"E, 1079 m a.s.l., on branches of *Castaneamollissima*, 1 Jul. 2017, N. Jiang, CFCC 52742 (BJFC-S1550).

##### Notes.

*Dendrostomashaanxiense*, *D.castaneae* and *D.castaneicola* are phylogenetically closely related species occurring on the same host, *Castaneamollissima* (Fig. 2). However, *Dendrostomashaanxiense* has conidia with a smaller l/w ratio than *D.castaneae* (3.3–4.2 in *D.shaanxiense* vs. 4.2–5.2 in *D.castaneae*) and has narrower conidia than *D.castaneicola* (2.5–3.1 μm diam. in *D.shaanxiense* vs. 3.2–3.8 μm diam. in *D.castaneicola*).

#### Dendrostoma shandongense

Taxon classificationFungiDiaporthalesErythrogloeaceae

C.M. Tian & N. Jiang sp. nov.

MB826826

Figure 12

##### Diagnosis.

*Dendrostomashandongense* is distinguished from its closest relative *D.chinensis* by the colour of conidiomata.

##### Holotype.

CHINA. Shandong Province: Rizhao City, Donggang District, chestnut plantation, 35°42'28"N, 119°46'23"E, 452 m a.s.l., on branches of *Castaneamollissima*, 14 Apr. 2017, N. Jiang (holotype: BJFC-S1567; ex-type culture: CFCC 52759).

##### Etymology.

*Shandongense*, referring to the Shandong Province in China.

##### Description.

*Sexual morph* not observed. *Asexual morph*: *Conidiomata* pycnidial, spherical, occurring separately, reddish-orange, semi-immersed in bark, 250–400 μm high, 450–650 μm diam.; wall of several layers of black *textura angularis*. *Conidiophores* reduced to conidiogenous cells. *Conidiogenous cells* lining the inner walls of cavity, hyaline, smooth, ampulliform, 6.5–13 × 1–2.5 μm. *Conidia* hyaline, aseptate, smooth, multiguttulate, thin-walled, fusoid to ellipsoid, apex acutely rounded, base truncate, (7.8–)8.1–8.8(–9) × (3.7–)3.8–4.3(–4.8) μm, l/w = (1.6–)1.9–2.3(–2.4) (n = 50).

**Figure 12.**
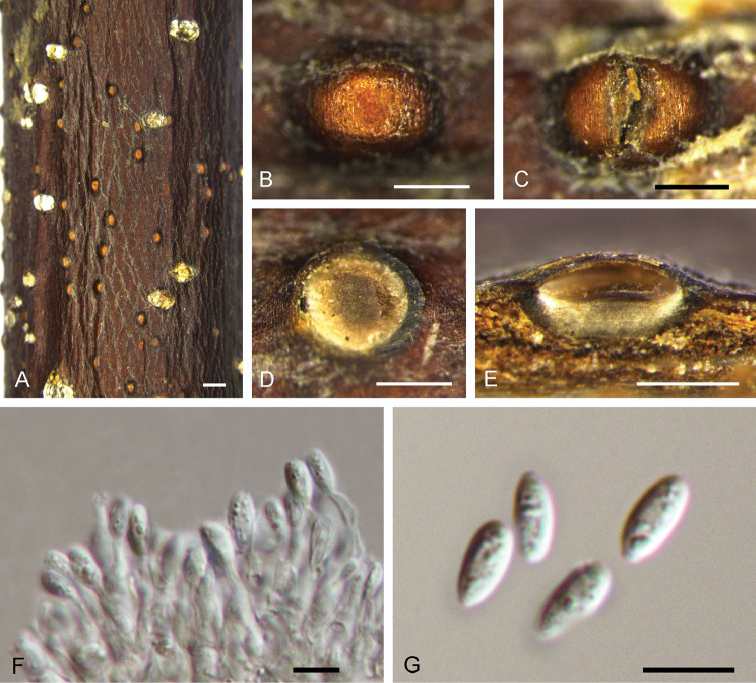
Morphology of *Dendrostomashandongense* from *Castaneamollissima* (BJFC-S1567). **A–C** Habit of conidiomata on branches **D** Transverse section of conidioma **E** Longitudinal section through conidioma **F** Conidiogenous cells **G** Conidia. Scale bars: 1 mm (**A**); 0.3 mm (**B–D**); 5 μm (**F**); 5 μm (**G**).

##### Culture characters.

On PDA, cultures are white. The colonies are flat with irregular edge; texture uniform, producing sparse conidiomata irregularly distributed near the centre of the plate within 1 month at 25 °C in the dark.

##### Additional specimen examined.

Shandong Province: Rizhao City, Donggang District, chestnut plantation, 35°42'28"N, 119°46'23"E, 452 m a.s.l., on branches of *Castaneamollissima*, 14 Apr. 2017, N. Jiang, CFCC 52760 (BJFC-S1568).

##### Notes.

*Dendrostomashandongense* and *D.chinensis* occasionally occur on the same branches. These species are best distinguished by the appearance of their conidiomata, which are black in *Dendrostomachinense* and orange in *D.shandongense*.

## Discussion

In this study, we reviewed the taxonomic circumscription of *Dendrostoma* using molecular and morphological data. This is the first study that presents a robust phylogeny using a number of *Dendrostoma* isolates from different geographic origins. The results revealed up to 14 species in *Dendrostoma* based on the observation of type specimens and ex-type cultures (*D.leiphaemia* was not observed), of which 10 species were shown to represent new species, namely *D.aurorae*, *D.castaneae*, *D.castaneicola*, *D.chinense*, *D.dispersum*, *D.parasiticum*, *D.qinlingense*, *D.quercus*, *D.shaanxiense* and *D.shandongense*.

**Figure 13.**
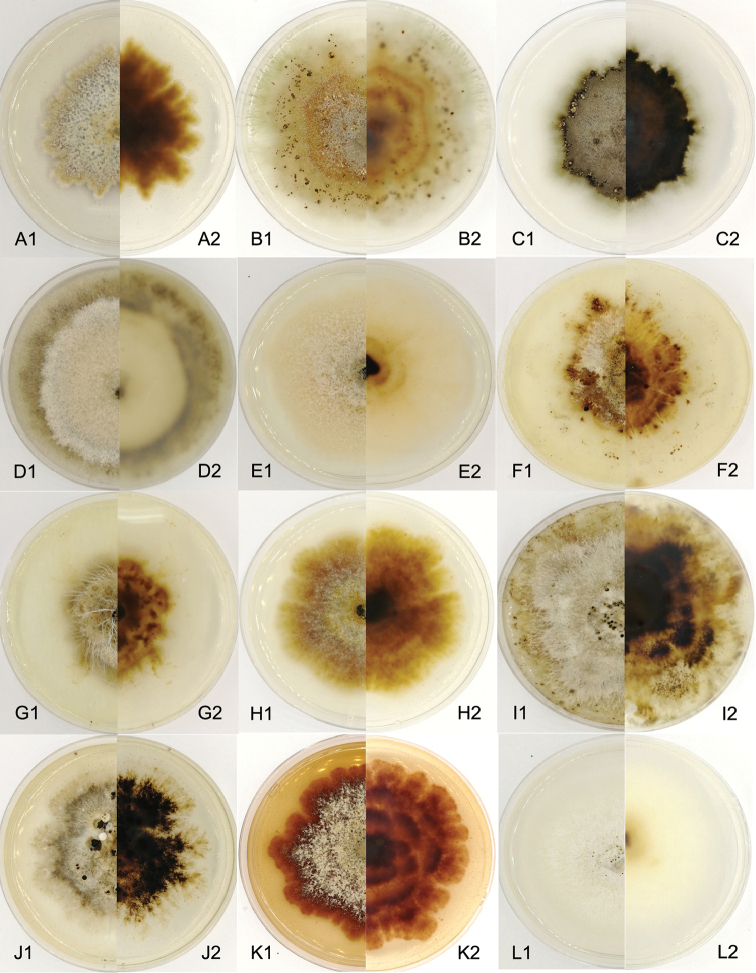
*Dendrostoma* cultures on PDA after 1 month at 25 °C, **A***D.aurorae***B***D.castaneae***C***D. castaneicola***D***D.chinense***E***D.dispersum***F–G***D.osmanthi***H***D.parasiticum***I***D.qinlingense***J***D.quercus*; **K***D.shaanxiense***L***D.shandongense*.

The 13 type specimens in *Dendrostoma* (except *D.leiphaemia*) were examined to establish robust morphological characteristics amongst specific ranks. Amongst these, 3 species, *Dendrostomamali*, *D.osmanthi* and *D.quercinum*, were discovered to only have a sexual morph on natural hosts; 9 species, *D.aurorae*, *D.castaneae*, *D. castaneicola*, *D.chinense*, *D.dispersum*, *D.parasiticum*, *D.qinlingense*, *D.shaanxiense* and *D.shandongense*, were observed with only an asexual morph and only one species, *D.quercus*, was represented by both asexual and sexual morphs. Hence, morphological differences amongst *Dendrostoma* species were mainly established based on conidiomata produced on diseased host tissues, including colours of conidiomata, culture characteristics (Fig. 13), existence or non-existence of a central column, conidial shape and dimensions.

*Dendrostomashandongense* and *D.chinense* are similar in conidial shape and size, but differ markedly from the other species. Additionally, *Dendrostomashandongense* and *D.chinense* comprise the only two species in the genus with conidiomata lacking a central column structure, although they differ considerably with regard to in conidiomatal appearance (Figs 6, 13). The remaining eight species differ by the existence of a central column inside the conidiomata and can be further distinguished by their conidial characteristics, namely length, width and l/w ratio. Additionally, a key to the 14 *Dendrostoma* species is provided below.

### Key to *Dentrostoma* species

**Table:** 

1	Asexual morphs with or without sexual morphs known from natural substrates	**2**
–	Only sexual morph known from natural substrates	**11**
2	Central column absent, length/width ratio of conidia < 3	**3**
–	Central column present, length/width ratio of conidia > 3	**4**
3	Conidiomata orange	*** D. shandongense ***
–	Conidiomata black	*** D. chinense ***
4	Conidia dimorphic	*** D. quercus ***
–	Conidia monomorphic	**5**
5	Conidial length > 15 μm	*** D. qinlingense ***
–	Conidial length < 15 μm	**6**
6	Conidial length/width ratio > 4.2	**7**
–	Conidial length/width ratio < 4.2	**8**
7	Conidial length/width ratio 4.2–5.2, conidial width 2.2–2.7 μm	*** D. castaneae ***
–	Conidial length/width ratio 4.9–5.9, conidial width 2–2.3 μm	*** D. dispersum ***
8	Central column white	*** D. castaneicola ***
–	Central column bright yellow or pale yellow	**9**
9	Central column pale yellow	*** D. aurorae ***
–	Central column bright yellow	**10**
10	Conidial width 2.8–3.3 μm, length/width ratio 3–3.9	*** D. parasiticum ***
–	Conidial width 2.5–3.1 μm, length/width ratio 3.3–4.2	*** D. shaanxiense ***
11	Ascospores width > 5 μm	*** D. leiphaemia ***
–	Ascospores width < 5 μm	**12**
12	Ascospores length > 15 μm	*** D. quercinum ***
–	Ascospores length < 15 μm	**13**
13	On *Osmanthus*, Ascospores 11.5–14.5 × 3.5–4 μm	*** D. osmanthi ***
–	On *Malus*, Ascospores 12–14 × 3–4 μm	*** D. mali ***


The genus *Dendrostoma* was initially proposed to include three presumed plant pathogens causing canker diseases on hardwood trees, namely *D.mali* on *Malusspectabilis*, *D.osmanthi* on *Osmanthusfragrans* and *D.quercinum* on *Quercusacutissima* (Fan et al. 2018). Consistent with the previous study, the newly described 10 species were all isolated from fruiting structures associated with typical canker symptoms on several hardwood tree species, namely *Castaneamollissima* and *Quercus* spp.

The tree genera *Castanea* and *Quercus* in Fagaceae contain numerous important and common tree species in China, including *C.mollissima*, *C.crenata*, *C.henryi*, *C.seguinii*, *Q.acutissima*, *Q.aliena*, *Q.dentata*, *Q.mongolica* and *Q.wutaishanica* (Flora of China website: http://frps.eflora.cn/). *Castaneamollissima* constitutes one the most important crop tree species widely cultivated in 26 provinces in China. However, many plantations and nurseries planting Chinese chestnut suffer from fungal diseases that cause high production losses (Jiang et al. 2018). In particular, chestnut blight caused by *Cryphonectriaparasitica* represents the most serious fungal disease, reducing host vitality and potentially killing the host (Jiang et al. 2018, Rigling and Prospero 2018).

In the present study, seven *Dendrostoma* species were observed on the host *Castaneamollissima* including *D.aurorae*, *D.castaneae*, *D.castaneicola*, *D.chinense*, *D. parasiticum*, *D.shaanxiense* and *D.shandongense*, causing chestnut canker diseases, termed Dendrostoma canker herein. Dendrostoma canker constitutes a newly discovered disease that has been observed in chestnut plantations and nurseries. Species of *Dendrostoma* usually infect host branches and stems, with occasional infection of twigs. Maturation of the fruiting structures from June to July resulted in death of the infected branches. Notably, no sexual fruiting structures were discovered during our investigations on chestnut trees.

Accurate recognition and identification of plant diseases are essential as fungal pathogens are constantly evolving and traditional control methods are frequently insufficient for disease control. In comparison, in the present study, Dendrostoma canker is considered to be caused by up to eight different species of *Dendrostoma*. Further studies are, however, required to confirm their pathogenicity and fully resolve their ecology.

## Supplementary Material

XML Treatment for Dendrostoma

XML Treatment for Dendrostoma aurorae

XML Treatment for Dendrostoma castaneae

XML Treatment for Dendrostoma castaneicola

XML Treatment for Dendrostoma chinense

XML Treatment for Dendrostoma dispersum

XML Treatment for Dendrostoma parasiticum

XML Treatment for Dendrostoma qinlingense

XML Treatment for Dendrostoma quercus

XML Treatment for Dendrostoma shaanxiense

XML Treatment for Dendrostoma shandongense

## References

[B1] Carbone I Kohn LM (1999) A method for designing primer sets for speciation studies in filamentous ascomycetes. Mycologia 91: 553–556. 10.2307/3761358

[B2] Crous PW Gams W Stalpers JA Robert V Stegehuis G (2004) MycoBank: an online initiative to launch Mycology into the 21^st^ century. Studies in Mycology 50: 19–22.

[B3] Crous PW Summerell BA Alfenas AC Edwards J Pascoe IG Porter IJ Groenewald JZ (2012a) Genera of diaporthalean coelomycetes associated with leaf spots of tree hosts. Persoonia 28: 66–75. 10.3767/003158512X642030 23105154 PMC3409416

[B4] Crous PW Summerell BA Shivas RG Burgess TI Decock CA Dreyer LL Granke LL Guest DI Hardy GE Hausbeck MK Hüberli D Jung T Koukol O Lennox CL Liew ECY Lombard L McTaggart AR Pryke JS Roets F Saude C Shuttleworth LA Stukely MJC Vánky K Webster BJ Windstam ST Groenewald JZ (2012b) Fungal Planet description sheets: 107–127. Persoonia 28: 138–182. 10.3767/003158512X652633 23105159 PMC3409410

[B5] Crous PW Wingfield MJ Burgess TI Hardy GSJ Barber PA Alvarado P Barnes CW Buchanan PK Heykoop M Moreno G Thangavel R van der Spuy S Barili A Barrett S Cacciola SO Cano-Lira JF Crane C Decock C Gibertoni TB Guarro J Guevara-Suarez M Hubka V Kolařík M Lira CRS Ordoñez ME Padamsee M Ryvarden L Soares AM Stchigel AM Sutton DA Vizzini A Weir BS Acharya K Aloi F Baseia IG Blanchette RA Bordallo JJ Bratek Z Butler T Cano-Canals J Carlavilla JR Chander J Cheewangkoon R Cruz RHSF da Silva M Dutta AK Ercole E Escobio V Esteve-Raventós F Flores JA Gené J Góis JS Haines L Held BW Jung MH Hosaka K Jung T Jurjević Ž Kautman V Kautmanova I Kiyashko AA Kozanek M Kubátová A Lafourcade M Spada FL Latha KPD Madrid H Malysheva EF Manimohan P Manjón JL Martín MP Mata M Merényi Z Morte A Nagy I Normand AC Paloi S Pattison N Pawłowska J Pereira OL Petterson ME Picillo B Raj KNA Roberts A Rodríguez A Rodríguez-Campo FJ Romański M Ruszkiewicz-Michalska M Scanu B Schena L Semelbauer M Sharma R Shouche YS Silva V Staniaszek-Kik M Stielow JB Tapia C Taylor PWJ Toome-Heller M Vabeikhokhei JMC Van Diepeningen AD Van Hoa N Van Tri M Wiederhold NP Wrzosek M Zothanzama J Groenewald JZ (2017) Fungal Planet description sheets: 558–624. Persoonia 38: 240–384. 10.3767/003158517X698941 29151634 PMC5645186

[B6] Crous PW Wingfield MJ Burgess TI Hardy GSJ Crane C Barrett S Cano-Lira JF Le Roux JJ Thangavel R Guarro J Stchigel AM Martín MP Alfredo DS Barber PA Barreto RW Baseia IG Cano-Canals J Cheewangkoon R Ferreira RJ Gené J Lechat C Moreno G Roets F Shivas RG Sousa JO Tan YP Wiederhold NP Abell SE Accioly T Albizu JL Alves JL Antoniolli ZI Aplin N Araújo J Arzanlou M Bezerra JDP Bouchara JP Carlavilla JR Castillo A Castroagudín VL Ceresini PC Claridge GF Coelho G Coimbra VRM Costa LA da Cunha KC da Silva SS Daniel R de Beer ZW Dueñas M Edwards J Enwistle P Fiuza PO Fournier J García D Gibertoni TB Giraud S Guevara-Suarez M Gusmão LFP Haituk S Heykoop M Hirooka Y Hofmann TA Houbraken J Hughes DP Kautmanová I Koppel O Koukol O Larsson E Latha KPD Lee DH Lisboa DO Lisboa WS López-Villalba Á Maciel JLN Manimohan P Manjón JL Marincowitz S Marney TS Meijer M Miller AN Olariaga I Paiva LM Piepenbring M Poveda-Molero JC Raj KNA Raja HA Rougeron A Salcedo I Samadi R Santos TAB Scarlett K Seifert KA Shuttleworth LA Silva GA Silva M Siqueira JPZ Souza-Motta CM Stephenson SL Sutton DA Tamakeaw N Telleria MT Valenzuela-Lopez N Viljoen A Visagie CM Vizzini A Wartchow F Wingfield BD Yurchenko E Zamora JC Groenewald JZ (2016) Fungal Planet description sheets: 469–557. Persoonia 37: 218–403. 10.3767/003158516X694499 28232766 PMC5315290

[B7] Doyle JJ Doyle JL (1990) Isolation of plant DNA from fresh tissue. Focus 12: 13–15.

[B8] Fan XL Bezerra JD Tian CM Crous PW (2018) Families and genera of diaporthalean fungi associated with canker and dieback of tree hosts. Persoonia 40: 119–134. 10.3767/persoonia.2018.40.05 30504998 PMC6146645

[B9] Ferreira FA Demuner NL Rezende DV (1992) Mancha de folha, des folha e antracnose do Jatobá (*Hymenaea* spp.) causadas por *Erythrogloeumhymenaeae*. Fitopatologia Brasileira 17: 106–109.

[B10] Guindon S Dufayard JF Lefort V Anisimova M Hordijk W Gascuel O (2010) New algorithms and methods to estimate maximum-likelihood phylogenies: assessing the performance of PhyML 3.0. Systematic Biology 59: 307–321. 10.1093/sysbio/syq010 20525638

[B11] Hillis DM Bull JJ (1993) An empirical test of bootstrapping as a method for assessing confidence in phylogenetic analysis. Systematic Biology 42: 182–192. 10.1093/sysbio/42.2.182

[B12] Jiang N Fan X Yang Q Du Z Tian CM (2018) Two novel species of *Cryphonectria* from *Quercus* in China. Phytotaxa 347: 243–250. 10.11646/phytotaxa.347.3.5

[B13] Katoh K Toh H (2010) Parallelization of the MAFFT multiple sequence alignment program. Bioinformatics 26: 1899–1900. 10.1093/bioinformatics/btq224 20427515 PMC2905546

[B14] Liu YJ Whelen S Hall BD (1999) Phylogenetic relationships among ascomycetes: evidence from an RNA polymerse II subunit. Molecular Biology and Evolution 16: 1799–1808. 10.1093/bioinformatics/btq224 10605121

[B15] Petrak F (1953) *Erythrogloeum* n. gen., eine neue Gattung der Sphaeropsideen. Sydowia 7: 378–380.

[B16] Rigling D Prospero S (2018) *Cryphonectriaparasitica*, the causal agent of chestnut blight: Invasion history, population biology and disease control. Molecular Plant Pathology 19: 7–20. 10.1111/mpp.12542 PMC6638123 28142223

[B17] Rossman AY Farr DF Castlebury LA (2007) A review of the phylogeny and biology of the Diaporthales. Mycoscience 48: 135–144. 10.1007/S10267-007-0347-7

[B18] Senanayake IC Crous PW Groenewald JZ Maharachchikumbura SS Jeewon R Phillips AJL Bhat JD Perera RH Li QR Li WJ Tangthirasunun N Norphanphoun C Karunarathna SC Camporesi E Manawasighe IS Al-Sadi AM Hyde KD (2017) Families of Diaporthales based on morphological and phylogenetic evidence. Studies in Mycology 86: 217–296. 10.1016/j.simyco.2017.07.003 28947840 PMC5603113

[B19] Senanayake IC Jeewon R Chomnunti P Wanasinghe DN Norphanphoun C Karunarathna A Pem D Perera RH Camporesi E McKenzie EHC Hyde KD Karunarathna SC (2018) Taxonomic circumscription of Diaporthales based on multigene phylogeny and morphology. Fungal Diversity 93: 241–443. 10.1007/s13225-018-0410-z

[B20] Tamura K Stecher G Peterson D Filipski A Kumar S (2013) MEGA6: Molecular Evolutionary Genetics Analysis version 6.0. Molecular Biology and Evolution 30: 2725–2729. 10.1093/molbev/mst197 24132122 PMC3840312

[B21] Vilgalys R Hester M (1990) Rapid genetic identification and mapping of enzymatically amplified ribosomal DNA from several *Cryptococcus* species. Journal of Bacteriology 172: 4238–4246. 10.1128/jb.172.8.4238-4246.1990 2376561 PMC213247

[B22] Voglmayr H Castlebury LA Jaklitsch WM (2017) *Juglanconis* gen. nov. on Juglandaceae, and the new family Juglanconidaceae (Diaporthales). Persoonia 38: 136–155. 10.3767/003158517X694768 29151630 PMC5645181

[B23] Voglmayr H Jaklitsch WM (2014) Stilbosporaceae resurrected: generic reclassification and speciation. Persoonia 33: 61–82. 10.3767/003158514X684212 25737594 PMC4312938

[B24] White TJ Bruns T Lee S Taylor JL (1990) Amplification and direct sequencing of fungal ribosomal RNA genes for phylogenetics. PCR Protocols: a guide to methods and applications 18: 315–322. 10.1016/B978-0-12-372180-8.50042-1

[B25] Zhang YJ Zhang S Liu XZ Wen HA Wang M (2010) A simple method of genomic DNA extraction suitable for analysis of bulk fungal strains. Letters in applied microbiology 51: 114–118. 10.1111/j.1472-765X.2010.02867.x 20536704

